# The Role for Dietary Omega-3 Fatty Acids Supplementation in Older Adults

**DOI:** 10.3390/nu6104058

**Published:** 2014-10-03

**Authors:** Alessio Molfino, Gianfranco Gioia, Filippo Rossi Fanelli, Maurizio Muscaritoli

**Affiliations:** 1 Department of Clinical Medicine Sapienza, University of Rome, Viale dell’Università 37, 00185 Rome, Italy; E-Mails: alessiomolfino@alice.it (A.M.); gioiag86@hotmail.it (G.G.); filippo.rossifanelli@uniroma1.it (F.R.F.)

**Keywords:** omega-3 fatty acids, eicosapentaenoic acid, docosapentaenoic acid, docosahexaenoic acid, older adults

## Abstract

Optimal nutrition is one of the most important determinants of healthier ageing, reducing the risk of disability, maintaining mental and physical functions, and thus preserving and ensuring a better quality of life. Dietary intake and nutrient absorption decline with age, thus increasing the risk of malnutrition, morbidity and mortality. Specific nutrients, particularly long-chain omega-3 polyunsaturated fatty acids (PUFAs), might have the potential of preventing and reducing co-morbidities in older adults. Omega-3 PUFAs are able to modulate inflammation, hyperlipidemia, platelet aggregation, and hypertension. Different mechanisms contribute to these effects, including conditioning cell membrane function and composition, eicosanoid production, and gene expression. The present review analyzes the influence of omega-3 PUFAs status and intake on brain function, cardiovascular system, immune function, muscle performance and bone health in older adults. Omega-3 FAs may have substantial benefits in reducing the risk of cognitive decline in older people. The available data encourage higher intakes of omega-3 PUFAs in the diet or via specific supplements. More studies are needed to confirm the role of omega-3 FAs in maintaining bone health and preventing the loss of muscle mass and function associated with ageing. In summary, omega-3 PUFAs are now identified as potential key nutrients, safe and effective in the treatment and prevention of several negative consequences of ageing.

## 1. Introduction

From 1935 to 2010, death rates decreased by 60% in the United States thus resulting in dramatic increases in the population of older adults from about 3 million at the turn of the 20th century to over 40 million in 2010. By the year 2050, the number of Americans 65 years or older is estimated to be around 88.5 million and 19 million will be 85 years or older. Public financing of long-term care will increase by 20%–21% by 2020 [1]. Malnutrition risk significantly increases with age, with subjects over 90 years being characterized by almost 40% higher malnutrition risk than those <70 years [2]. Dietary intake changes with age and the mean energy intake decline with age in both males and females [3], thus increasing morbidities. Optimal nutrition is one of the most important determinants of healthier ageing, reducing the risk of disability, maintaining mental and physical functions, thus preserving and ensuring a better quality of life [4,5]. Reduced nutrient consumption with age and age-related decline in absorptive and metabolic function contributes to the altered nutrient intake associated with ageing [6]. In fact, several factors may negatively impact on nutrient intake in older adults. These include socio-economic status and physical and mental health [7], while age does not affect bioavailability of exogenously supplemented lipids [8].

Specific nutrients, particularly long-chain omega-3 polyunsaturated fatty acids (PUFAs), might have the potential of preventing and reducing co-morbidities in older adults [9,10], including cognitive decline and cardiovascular events. Omega-3 PUFAs such as alpha-linolenic acid (ALA), eicosapentaenoic acid (EPA), docosapentaenoic acid (DPA), and docosahexaenoic acid (DHA) have been widely evaluated in epidemiological studies. Due to the limited ability for humans to elongate and desaturate alpha-linolenic acid to the long chain omega-3 PUFA [11], it is necessary to obtain adequate amounts through fish and fish-oil products that contain high levels of omega-3 PUFAs. Health benefits including cardio-protective effects such as anti-hyperlipidemia, anti-thrombotic, anti-inflammatory, anti-hypertensive, and anti-arrhythmic effects have been correlated with omega-3 PUFAs consumption [12,13]. The Omega-3 Index, *i.e.*, the EPA + DHA content of erythrocyte membranes, expressed as a percentage of total identified FAs, is nowadays used as a marker for coronary heart disease (CHD) risk, its optimal levels appearing to be 8% or greater [14,15]. An Omega-3 Index ≥8% was associated with the greatest cardioprotection, whereas an index ≤4% was associated with the least [16]. Based on these premises, omega-3 FAs are now generally recognized as potential key nutrients to promote healthier ageing [17]. The focus of the present paper is to review the impact of omega-3 FAs on cognitive function, cardiovascular system, bone health, muscle function and immune response in older adults. We examined studies involving subjects >65 years in which omega-3 PUFAs status and intake (from natural food sources and/or supplements) were correlated with clinical outcomes.

## 2. Effects of Omega-3 PUFAs on Brain Function

### 2.1. Dementia

The levels of EPA, DHA, and total omega-3 PUFAs are significantly decreased in peripheral blood tissues of patients affected by dementia [18]. DHA, which is particularly represented in the brain, is neuroprotective and contributes to normal brain function. Dietary DHA content and hepatic conversion from dietary derived ALA determine its brain concentration as confirmed by an *in vivo* experimental model in which the brain incorporation rate of DHA is equal to the brain consumption rate of DHA [19]. Higher plasma EPA levels were coupled with a decreased incidence of dementia in a cohort of 1214 older non-demented persons followed up for 4 years, independently of depressive status; moreover, increased ratios of omega-6 to omega-3 FAs and AA to DHA were associated with an augmented risk of dementia, also in older age depressive status [20]. Nine hundred mg/day of DHA supplementation for 24 weeks ameliorated memory in healthy older adults with age-related cognitive decline without side effects. In particular, DHA administration was associated with improvement in immediate and delayed verbal recognition memory scores, and Paired Associate Learning (PAL) scores [21]. A 12-month fish oil (FO) supplementation with concentrated DHA in 36 low-socioeconomic-status elderly subjects with mild cognitive impairment (MCI) significantly ameliorated short-term and working memory, immediate verbal memory and delayed recall capability. The 12-month change in memory also significantly improved after treatment with good tolerance and minimal side effects [22]. Eight hundred mg/day of DHA or/and 12 mg/day of lutein supplementation for 4 months significantly enhanced verbal fluency scores in unimpaired elder women. Memory scores and rate of learning significantly increased after the combined supplementation, without influencing mental processing speed, accuracy and mood [23]. The administration of an oily emulsion of DHA-phospholipids containing melatonin and tryptophan for 12 weeks in 25 elderly subjects with MCI led to a significant treatment effect for the Mini-Mental State Examination (MMSE) and the olfactory sensitivity assessment, a positive trend for the semantic verbal fluency, and a significant improvement in the Mini Nutritional Assessment (MNA) score [24].

### 2.2. Depression and Cognitive Function

EPA and DHA supplementation for 6 months in 50 people aged >65 years with MCI showed improvement in Geriatric Depression Scale (GDS) scores and mental health while verbal fluency and self-reported physical health ameliorated only in the DHA group [25]. Three g/day of FO omega-3 PUFAs supplementation for 5 weeks in 40 healthy middle aged to elderly subjects led to enhanced cognitive performance in the working memory test, lowered plasma triacylglycerides and lowered systolic blood pressure [26]. The administration of phosphatidylserine (PS) containing omega-3 long-chain PUFAs attached to its backbone (PS-DHA) for 15 weeks in non-demented elderly with memory complaints may significantly improve cognitive performance parameters such as immediate and delayed verbal recall, time to copy complex figures and learning abilities, especially participants with higher baseline cognitive status. Efficacy measures in this study were a computerized cognitive battery, the Rey Auditory Verbal Learning Test, and the Rey Complex Figure Test [27]. The daily supplementation for 3 years of the combination of antioxidants omega-3 PUFAs, lycopene, and Ginkgo biloba extracts synergistically improved cognitive function of 41 subjects from a community dwelling aged 65 years and older in both apolipoprotein E (APOE4) non-carrier (E4-) and APOE4 carrier (E4+) groups during a 3-year follow-up without any influence of (APOE) genotype on the effect of antioxidants [28]. A similar pattern in omega-3 dietary intake and the percentage of EPA and DHA in blood plasma phosphatidylcholine (PC) was described in individuals with cognitive impairment no dementia (CIND), individuals with Alzheimer’s disease (AD), and healthy volunteers (HV) aged between 55 and 91 years within a hospital setting (AD < CIND < HV). Moreover, plasma PC DHA, plasma PC EPA, and omega-3 intake positively predicted memory functioning. Dietary omega-3 FAs changes also consequent to cognitive derangements probably influence cognitive decline [29]. No significant differences were found between supplemented and placebo groups over 24 months in the California Verbal Learning Test (CVLT), cognitive function scores and secondary cognitive outcomes after daily administration of capsules containing 200 mg EPA plus 500 mg DHA for 24 months to cognitively healthy older people aged 70 years or older. The ability to check on any potential benefit of FO on cognitive function was limited by the lack of decline in cognitive function in either study arms [30]. No overall effect of 1800 mg/day of EPA-DHA or 400 mg/day of EPA-DHA supplementation for 26 weeks on cognitive performance was observed in cognitively healthy individuals aged 65 years or older taking into account memory, executive function, cognitive domains of attention, and sensory motor speed [31]. Lowered severity of depressive symptomatology, evaluated by the Center for Epidemiologic Studies Depression scale, was coupled with higher plasma EPA levels in French elderly community dwellers, particularly those receiving antidepressants [32]. No effect of 1800 or 400 mg/day EPA + DHA supplementation for 26 weeks in independently living individuals aged 65 years or older was reported on mental well-being, assessed with the Center for Epidemiologic Studies Depression Scale, Montgomery-Asberg Rating Scale, Geriatric Depression Scale, and Hospital Anxiety and Depression Scale [33]. In summary, as shown in Table 1 (nine positive studies, seven controlled trials), the majority of the available evidence supports a positive correlation between omega-3 status/supplementation with brain function.

## 3. Effects of Omega-3 PUFAs on Cardiovascular System

### 3.1. Atherosclerosis

Omega-3 to omega-6 PUFA ratio may influence cardiovascular events as shown in a recent study comparing the influences of omega-3 to omega-6 PUFA ratios on coronary atherosclerosis, evaluated by virtual histology intravascular ultrasound, in non-culprit lesions in the percutaneous coronary intervention vessel in patients treated with statins for 8 months [34]. The percentage change in plaque volume was negatively correlated to the changes in the DHA/AA ratio and EPA + DHA/AA ratio in the pravastatin group. The development of coronary atherosclerosis is associated with decreased omega-3 to omega-6 PUFAs ratios following pravastatin therapy [34]. Six weeks of supplementation with EPA plus DHA from FO in addition to the best medical therapy, such as aspirin and statin therapy, resulted in no effects on inflammation and platelet and endothelial activation in patients affected by peripheral arterial disease. In fact, no modulation on von Willebrand factor, P-selectin expression, fibrinogen binding, platelet aggregation, pulse wave velocity, high-sensitivity C-reactive protein, s-ICAM and IL-6 [35] was observed. There is a difference in phospholipid FAs composition between non-stroke patients and ischemic stroke patients and also between no cerebral atherosclerotic stenosis (NCAS) and intracranial atherosclerotic stenosis (ICAS) in strokes. In particular, DHA and EPA distinguish NCAS and ICAS. The risk of ICAS may be increased at lower amounts of DHA in plasma or in diet and is inversely related to phospholipid DHA levels [36].

### 3.2. Arrhythmias

Supplementation with 1 g/day of omega-3 PUFAs for 6 months in patients with history of myocardial infarction was able to reduce the number of isolated and paired premature ventricular contractions and the number of unstable ventricular tachycardia paroxysms, thus enhancing the effect of antiarrhythmic therapy. In addition, omega-3 PUFAs improve heart rate variability and increased red blood cells’ omega-3 index [37].

Although a study population including adults aged 65 or older, not affected by atrial fibrillation and coronary heart disease, showed no association of dietary ALA and plasma phospholipids with incident atrial fibrillation [38], the supplementation of omega-3 PUFAs, coupled with the antioxidant Vitamins C and E in patients over 60 years old scheduled for cardiac surgery with extracorporeal circulation enhanced glutathione peroxidase activity in atrial tissue and decreased the incidence of postoperative atrial fibrillation [39].

**Table 1:** Summary of trials evaluating the effects of omega-3 FAs in older adults.

Function	Summary of Positive Effects	Reference(s)
Cognitive (9 positive studies, 3 negative studies)	Incidence of dementia ↓	[20]
	Immediate and delayed verbal recognition memory scores ↑	[21]
	PAL score ↑	[21]
	Short-term and working memory ↑	[22]
	Immediate verbal memory ↑	[22]
	Delayed recall capability ↑	[22]
	12-month change in memory ↑	[22]
	Verbal fluency scores ↑	[23]
	Memory scores ↑	[23]
	Rate of learning ↑	[24]
	MMSE ↑	[24]
	Olfactory sensitivity assessment ↑	[24]
	Semantic verbal fluency ↑	[24]
	MNA score ↑	[25]
	GDS scores ↑	[25]
	Mental health ↑	[25]
	Verbal fluency ↑	[25]
	Working memory test ↑	[26]
	Immediate and delayed verbal recall ↑	[27]
	Time to copy complex figure ↓	[27]
	Learning abilities ↑	[27]
	Depressive symptomatology ↓	[32]
Cardiovascular (9 positive studies, 3 negative studies)	Plasma tryacilglycerydes ↓	[26]
	Systolic blood pressure ↓	[26]
	Coronary atherosclerosis plaque volume ↓	[34]
	Risk of ICAS ↓	[36]
	Isolated and paired premature ventricular contractions ↓	[37]
	Unstable ventricular tachycardia paroxysms ↓	[37]
	The effect of antiarrhythmic therapy ↑	[37]
	Hearth rate variability ↑	[37,40]
	Red blood cells omega-3 index ↑	[37]
	Glutathione peroxidase activity in atrial tissue ↑	[39]
	Incidence of postoperative atrial fibrillation ↓	[39]
	Cardiac autonomic modulation ↑	[41]
	HF-related total mortality ↓	[41]
	Mean RR interval ↑	[41]
	Standard deviation of all normal-to-normal RR intervals ↑	[41]
	Turbulence slope ↑	[41]
	Very low frequency power ↑	[41]
	Incident CHF risk ↓	[42]
	Percentage of successive normal RR intervals differing by more than 50 ms ↑	[40]
	EPA and DHA in platelet and atrial tissue membranes ↑	[43]
Immune (7 positive studies, no negative studies)	Proliferative response of T lymphocytes ↑	[44]
	Serum IL-10 ↑	[45]
	Serum Tumor necrosis factor-α ↓	[45]
	Serum IL-8 ↓	[45]
	Inflammation ↓	[45]
	Perioperative systemic inflammation ↓	[43]
	Postoperative IL-6 ↓	[43]
	Lymphocyte proliferation ↓	[46,47]
	Lymphocyte particulate phosphodiesterase activity↓	[47]
	Glutathione peroxidase activity ↓	[47]
	Natural killer cell activity ↓	[48]
	Prostaglandin E2 production by mononuclear cells ↓	[49]
	Neutrophil respiratory burst ↓	[49]
Bone & Muscle (6 positive studies, no negative studies)	Muscle protein synthesis ↑	[50]
	BMD ↑	[51,52]
	Knee flexor muscle thickness ↑	[53]
	IL-6 during resistance training ↓	[53]
	Grip strength ↑	[54]
	Lower extremity performance ↑	[55]
	Physical performance ↑	[55]
Other (2 positive studies, 1 negative study)	Plasma glucose, lactate, blood carboxyhemoglobin after surgery ↓	[43]
	Serum adiponectin ↑	[56]

PAL (Paired Associate Learning), Mini-Mental State Examination (MMSE), Mini Nutritional Assessment (MNA), Geriatric Depression Scale (GDS), Intracranial atherosclerotic stenosis (ICAS), Hearth failure (HF), Congestive heart failure (CHF), Bone mineral density (BMD).

Supplementation of 1 g/day of omega-3 PUFAs for 3 months partially improved cardiac autonomic regulation in patients affected by chronic heart failure (HF) with a left ventricular ejection fraction <40%. Results consisted in an increase in mean RR interval, standard deviation of all normal-to-normal RR intervals, turbulence slope, and very low frequency power after 3 months of supplementation [41]. Plasma phospholipid EPA concentration in older adults without prevalent heart disease enrolled in the Cardiovascular Health Study from 1992 to 2006 was inversely associated with incident congestive heart failure risk while circulating DPA and total long-chain omega-3 FAs concentrations were associated with a trend toward lower risk [42]. Low-dose ALA, EPA-DHA, and EPA-DHA plus ALA supplementation did not affect serum total testosterone levels and the risk of incident testosterone deficiency in post-myocardial infarction (MI) male patients aged 60–80 years [57]. Supplementation with 2 g/day of FO for 5 months prevented heart rate variability decline in nursing home residents older than 60 years [40]. Based on the reviewed literature, nine out of 12 studies indicate that omega-3 status/supplementation positively impacts on cardiovascular function in older adults (Table 1).

## 4. Effects of Omega-3 PUFAs on Immune Function

### 4.1. Immune Cell Proliferation

The essential FAs are known to modulate T cell proliferation and inflammatory responses as shown in older adults consuming novel soybean oils varying in LA:ALA ratios for 35 days [44]. The LA:ALA ratio modulates the proliferative ability of T lymphocytes probably in consequence of changes in FAs composition of the phospholipids in immune cells with an optimal proliferative response at an LA:ALA ratio of 8.70 corresponding to soybean oil diet. In particular, stronger proliferative responses to phytohemagglutinin were shown following consumption of soybean oil, low-saturated fatty acid (SFA) soybean oil or high-oleic acid soybean oil diets. Instead, proliferative response was similar to the baseline after consuming hydrogenated soybean oil and low-ALA soybean oil diets with an LA:ALA ratio >10 [44].

### 4.2. Pro-Inflammatory Cytokines

Short-term intravenous administration of FO-based lipid emulsion (0.2 g/kg body weight) over 6 h for 3 consecutive days in critically ill enterally fed elderly patients in the first 48 h of intensive care unit (ICU) admission increased energy intake and serum IL-10 concentration, decreased serum tumor necrosis factor-α and IL-8 concentrations, occurring around 7–9 days of ICU stay, thereby expressing an anti-inflammatory effect [45]. Three perioperative infusions of 0.2 g/kg FO emulsion 12 and 2 h before and immediately after surgery significantly increased EPA and DHA concentrations in platelet and atrial tissue membranes within 12 h of the first supplementation and decreased systemic inflammation induced by a cardiopulmonary bypass thus suggesting benefits in elective cardiac surgery patients. In particular, FO administration significantly decreased the postoperative increase in the IL-6 and lowered plasma glucose, lactate, and blood carboxyhemoglobin on the day after surgery without any adverse effect [43]. A clinical study analyzed the effects of dietary supplementation for 12 weeks with moderate levels of ALA, gamma-linolenic acid (GLA), AA, DHA or FO [2 g/day of ALA or 770 mg/day of GLA or 680 mg/day of AA or 720 mg/day of DHA or 1 g/day of EPA plus DHA (720 mg of EPA + 280 mg of DHA)] on the proliferation of mitogen-stimulated human peripheral blood mononuclear cells (PBMC) and the production of cytokines by those cells in healthy subjects. GLA, AA, DHA and FO supplementation changed the FA composition of PBMC phospholipids. However, ALA, AA or DHA treatments did not affect lymphocyte proliferation. Instead, GLA and FO supplementation significantly decreased lymphocyte proliferation. The production of IL-2, interferon-gamma by PBMC and the number of circulating T or B lymphocytes, helper or cytotoxic T lymphocytes or memory helper T lymphocytes were not affected by these treatments [46].

### 4.3. Other Cellular Effects

The supplementation of healthy elderly people with 600 mg/day of marine oil, providing 150 mg DHA plus 30 mg EPA for 6 weeks, significantly decreased the proliferative responses of lymphocytes to mitogens at day 42 and a slight reduction of their cytosolic cyclic nucleotide phosphodiesterase (PDE) activity, a marked increase of their particulate PDE activity, a slight increase in cyclic nucleotide intracellular levels, and a depressed glutathione peroxidase activity was demonstrated [47]. Healthy subjects aged 55–75 years in these groups consumed 2 g ALA, 770 mg GLA, 680 mg AA, 720 mg DHA, or 720 mg EPA plus 280 mg DHA daily, respectively, for 12 weeks in the form of oils. The FA composition of plasma phospholipids changed after GLA, AA, DHA, and FO supplementation. The placebo, ALA, GLA, AA, or DHA treatments did not influence natural killer (NK) cell activity while FO administration significantly reduced NK cell activity. It was therefore concluded that moderate amounts of EPA can reduce NK cell activity [48]. The consumption of different amounts of an oil providing 1.35, 2.7, or 4.05 g EPA/d for 12 weeks in healthy young and older men was associated with increased incorporation of EPA into plasma or mononuclear cells (MNC) phospholipids and decreased production of prostaglandin E2 by MNC. Nevertheless, EPA supplementation decreased neutrophil respiratory burst only in older men in a dose-dependent manner [49]. The available data suggest that immune function is significantly modulated by omega-3 FA status/supplementation.

## 5. Effects of Omega-3 PUFAs on Muscle Mass and Function

Procatabolic hormonal milieu, cytokine milieu, age and immobility negatively influence the maintenance of muscle protein synthesis. Ageing is associated with reduced ability to capture blood-borne amino acids as protein, decreased protein synthesis, a decrease in signaling proteins capacity to indicate the presence of amino acids, and resistance to the effects of insulin in reducing muscle proteolysis and improving muscle anabolic response. All of the above factors contribute to the onset of anabolic resistance [58]. The progressive loss of muscle mass and function, *i.e.*, sarcopenia occurs with ageing. Approximately 1%–2% of muscle mass per year is lost after the age of 50. The age-related reduction in muscle mass and strength is accompanied by intramuscular fat accumulation, muscle atrophy, decreased satellite cell proliferation and differentiation capacity, and reduction in motor unit number. Sarcopenia of older adults represents an onerous healthcare cost: in the United States about 1.5% of total healthcare costs were attributable to sarcopenia in 2000 [59]. The stimulation of muscle protein synthesis induced by omega-3 FAs supplementation might be useful for the treatment and prevention of sarcopenia of ageing. Omega-3 FAs supplementation for 8 weeks in 16 older adults intensified the hyperaminoacidemia-hyperinsulinemia-induced increase in the rate of muscle protein synthesis together with greater increases in muscle mTOR and p70s6k phosphorylation [50]. The dietary intake of omega-3 FAs in older adults may be insufficient. Higher omega-3 FAs mean intake (1.27 g/day) was associated with higher bone mineral density (BMD) in a cross-sectional analysis involving subjects. No independent association was shown between omega-3 FA intake and lower extremity muscular function [51]. Fourteen g/day of ALA supplementation in the form of flaxseed oil for 12 weeks in older adults during a resistance training program lowered IL-6 concentration in males but not in females, together with a minimal effect on muscle mass and strength. ALA supplementation determined a greater increase in knee flexor muscle thickness in males [53]. A cross-sectional and retrospective cohort study analyzed the relationship between diet and grip strength in older adults and whether prenatal growth influences this relationship. Grip strength was inversely associated with age and positively related with height and weight at birth. The consumption of each additional portion of fatty fish per week determined an increase in grip strength of 0.43 kg in men and 0.48 kg in women, independently of adult height, age, and birth weight. These results confirm the role of omega-3 FAs in the prevention of sarcopenia [54]. A population-based study of older Italians enrolled 330 participants with impaired lower extremity performance (SPPB score < or =9). Higher levels of total PUFAs, omega-3 PUFAs, and omega-6 PUFAs were associated with a SPPB score >9. Participants with a SPPB score >9 had lower levels of SFA than those with a SPPB score <9. Impaired lower extremity performance (SPPB < or =9) was developed in 12.9% of the participants with a SPPB score >9 at baseline. Baseline omega-3 PUFAs plasma levels, reflecting dietary intake, were inversely associated with the risk of developing a reduction in SPPB to < or =9 and seem to protect against decline of physical function. Higher omega-6/omega-3 ratio was related to higher risk of SPPB decline to < or =9 and higher risk of developing poor physical performance [55]. It is concluded that omega-3 FA status and supplementation are strongly correlated with maintenance of muscle mass and function in older adults.

## 6. Effects of Omega-3 PUFAs on Bone Health

A higher dietary ratio of omega-6 to omega-3 FAs was associated with lower hip bone mineral density (BMD) in 1532 community-dwelling subjects aged 45–90 years. The ratio of dietary LA to ALA was inversely associated with hip BMD, independently of hormone therapy. A higher ratio of total dietary omega-6 to omega-3 FAs was also associated with lower BMD at the spine in women not undergoing hormone therapy and at the hip in all women [52]. In summary, bone health is significantly correlated with omega-3 status.

## 7. Other Effects of Omega-3 PUFAs

The transcription factor peroxisome proliferator-activated receptor-γ (PPARγ), which, among other functions, regulates the expression of adiponectin, is activated by marine omega-3 PUFAs administration. A study analyzed the interactions between dietary omega-3 PUFAs and adiponectin gene single nucleotide polymorphism (SNP) genotypes. Serum adiponectin was measured in healthy subjects aged 45–70 years supplemented with 0.45, 0.9, and 1.8 g/day of EPA plus DHA for 12 months. Subjects aged >58 years taking the highest dose presented a significant interaction between treatment and age in determining adiponectin. An association between a higher serum adiponectin concentration at baseline and the −11391 A-allele at the ADIPOQ locus was shown [56]. The highest dose administered to individuals homozygous for the +45 T-allele aged >58 years determined a 22% increase in serum adiponectin levels in comparison with baseline values. Older individuals, particularly those with the +45 TT genotype and reported increased risk for hypoadiponectinemia, type 2 diabetes, and obesity, may have benefits from a diet rich in omega-3 PUFAs [56]. The Carotenoids in Age-Related Eye Disease Study recruited women aged 50–79 years with high and low lutein intake in order to find relationships between the amount and type of dietary fat and intermediate age-related macular degeneration (AMD). Omega-6 and omega-3 PUFA intakes were associated with higher prevalence of intermediate AMD while monounsaturated fatty acids (MUFAs) intake was associated with lower prevalence. An association between total fat and saturated fatty acid intakes and increased prevalence of AMD in women aged <75 years in opposition to an inverse association in older women was reported [60]. Diets high in MUFAs were associated with lower prevalence of AMD in the whole population, thereby being protective [60].

**Figure 1:**
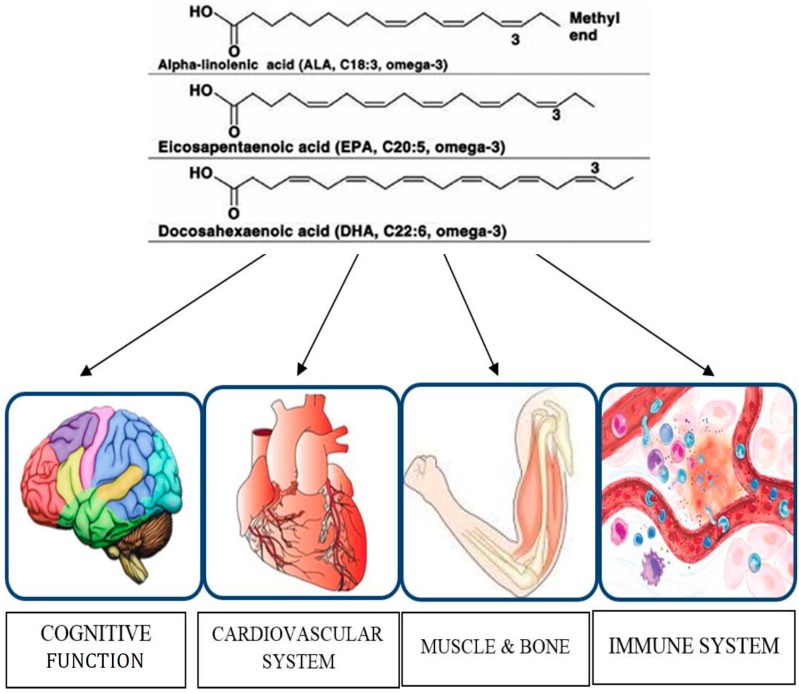
Organs and functions modulated by omega-3 PUFAs in older adults.

## 8. Conclusions

This review gathers all the most relevant recent clinical trials on omega-3 PUFAs supplementation in older adults. In particular, we examined the effects of omega-3 FAs on different clinical outcomes, namely cognitive decline, cardiovascular system, bone health, muscle performance and immune function, as shown in Figure 1. The literature review suggests that the omega-3 FAs may have substantial benefits in reducing the risk of cognitive decline in older people [61]. The available data encourage higher intakes of omega-3 PUFAs in the diet or specific supplements, but more studies are needed to clarify the conflicting results in the literature on the effectiveness of omega-3 FAs in maintaining bone health and preventing the loss of muscle mass and function associated with physiological ageing. Immune function declines with age and older adults are more susceptible to infections. These ageing deficiencies can be a consequence of derangements in food intake, nutrient absorption and metabolism of nutrients, but also to alterations associated with normal ageing. Omega-3 FAs may affect the immune response in older adults under dietary supplementation, but the available studies have not evaluated the long-term effects and the modification of immunological biomarkers after supplementation is stopped. Longer-term studies are needed to identify greater changes in study participants due to the effects of omega-3 PUFAs supplementation and to establish definitive influences on quality of life [62]. No significant side effects were reported after omega-3 PUFAs administration, confirming the safety of using these nutritional supplements. Finally, earlier intervention might prove useful in potentially treating age-related memory disorders and maintaining cognitive function, muscle performance and immune function during ageing. Research in this respect is therefore worthwhile.
